# Point-of-care ultrasound (POCUS) protocol for systematic assessment of the crashing neonate—expert consensus statement of the international crashing neonate working group

**DOI:** 10.1007/s00431-022-04636-z

**Published:** 2022-10-14

**Authors:** Yasser Elsayed, Muzafar Gani Abdul Wahab, Adel Mohamed, Nadya Ben Fadel, Shazia Bhombal, Nadya Yousef, María V. Fraga, Jehier Afifi, Pradeep Suryawanshi, Abbas Hyderi, Anup Katheria, Martin Kluckow, Daniele De Luca, Yogen Singh

**Affiliations:** 1 Section of Neonatology, Department of Pediatrics, University of Manitoba, Winnipeg, MB Canada; 2 Section of Neonatology, Department of Pediatrics, McMaster University, Hamilton, Canada; 3 Department of Pediatrics, University of Toronto, Toronto, Canada; 4 Department of Pediatrics, University of Ottawa, Ottawa, Canada; 5 Department of Pediatrics, Division of Neonatal and Behavioral Medicine, Stanford University School of Medicine, Palo Alto, CA USA; 6 Division of Pediatrics and Neonatal Critical Care, “A. Béclère” Medical Centre, APHP - Paris Saclay University Hospitals, Paris, France; 7 Department of Pediatrics, Division of Neonatology, Children’s Hospital of Philadelphia and Perelman School of Medicine, Philadelphia, USA; 8 Department of Pediatrics, Division of Neonatal Perinatal Medicine, Dalhousie University, Halifax, NS Canada; 9 Department of Neonatology, Bharati Vidyapeeth University Medical College, Pune, Maharashtra India; 10 Department of Pediatrics, Division of Neonatology, University of Alberta, Edmonton, Canada; 11 Department of Neonatology, Sharp Mary Birch Hospital for Women & Newborns, San Diego, CA USA; 12 Department of Neonatology, Royal North Shore Hospital and University of Sydney, Sydney, Australia; 13 Physiopathology and Therapeutic Innovation Unit-INSERM U999, Paris Saclay University, Paris, France; 14 Department of Pediatrics, Division of Neonatology, School of Clinical Medicine, Loma Linda University, Loma Linda University Children’s Hospital, Campus Street Coleman Pavillion, Loma Linda, CA 11175 USA; 15 Cambridge University Hospitals NHS Foundation Trust, Cambridge, UK

**Keywords:** Crashing infant, Point-of-care ultrasound (POCUS), Crashing Neonate Protocol (CNP), Collapsed neonate, Collapsing neonate, Neonate

## Abstract

Sudden unexpected clinical deterioration or cardiorespiratory instability is common in neonates and is often referred as a “crashing” neonate. The established resuscitation guidelines provide an excellent framework to stabilize and evaluate these infants, but it is primarily based upon clinical assessment only. However, clinical assessment in sick neonates is limited in identifying underlying pathophysiology. The Crashing Neonate Protocol (CNP), utilizing point-of-care ultrasound (POCUS), is specifically designed for use in neonatal emergencies. It can be applied both in term and pre-term neonates in the neonatal intensive care unit (NICU). The proposed protocol involves a stepwise systematic assessment with basic ultrasound views which can be easily learnt and reproduced with focused structured training on the use of portable ultrasonography (similar to the FAST and BLUE protocols in adult clinical practice). We conducted a literature review of the evidence-based use of POCUS in neonatal practice. We then applied stepwise voting process with a modified DELPHI strategy (electronic voting) utilizing an international expert group to prioritize recommendations. We also conducted an international survey among a group of neonatologists practicing POCUS. The lead expert authors identified a specific list of recommendations to be included in the proposed CNP. This protocol involves pre-defined steps focused on identifying the underlying etiology of clinical instability and assessing the response to intervention.

*Conclusion*: To conclude, the newly proposed POCUS-based CNP should be used as an adjunct to the current recommendations for neonatal resuscitation and not replace them, especially in infants unresponsive to standard resuscitation steps, or where the underlying cause of deterioration remains unclear.

**Table:** 

**What is known?**
*• Point-of-care ultrasound (POCUS) is helpful in evaluation of the underlying pathophysiologic mechanisms in sick infants.*
**What is new?**
*• The Crashing Neonate Protocol (CNP) is proposed as an adjunct to the current recommendations for neonatal resuscitation, with pre-defined steps focused on gaining information regarding the underlying pathophysiology in unexplained “crashing” neonates.*
*• The proposed CNP can help in targeting specific and early therapy based upon the underlying pathophysiology, and it allows assessment of the response to intervention(s) in a timely fashion.*

## Introduction

Point-of-care ultrasound (POCUS) refers to ultrasonography done at the bedside by the clinician caring for the patient [1]. It is performed in real time, with serial assessments longitudinally as required, to monitor disease progress and evaluate the response to interventions [2]. POCUS is a clinical tool applied to answer a practical specific question and guide critical care interventions, rather than a substitute for medical imaging performed and interpreted by diagnostic specialists (such as pediatric radiologists or cardiologists) [3]. A Crashing Neonate Ultrasound Protocol (CNP) could be used to assess any newborn needing or likely to need critical care, especially if the underlying cause is unknown. The routine use of POCUS has been suggested in clinical situations where the underlying mechanism of deterioration is unclear [3], and some authors have already proposed an algorithm for assessing life-threatening events in neonates admitted to the neonatal intensive care unit (NICU) such as SAFE/SAFER (Sonographic Assessment of Life-Threatening Events-Revised) protocol [4, 5].

A specific international working group of experts in POCUS was created to develop a screening protocol incorporating a quick bedside multiorgan ultrasound evaluation to understand the underlying mechanism of deterioration in a critically unwell newborn. The CNP protocol represents an expert consensus by POCUS key leaders, built on appropriate methodology, for the use of POCUS applications in the critically ill or crashing neonate in NICU. The proposed CNP is specifically designed for use in neonatal emergencies leading to significant cardiorespiratory instability and can be used in both term and pre-term neonates who are either “crashed” (needing resuscitation) or “crashing” (likely to need resuscitation if not stabilized soon) infants. CNP proposes a stepwise systematic targeted assessment with simple basic ultrasound views which are easily reproducible and can be learnt with a focused training, similar to the already established SAFE/SAFER in the newborn [5]. The CNP introduces a new approach with pre-defined steps focusing at assessing the underlying etiology for unresponsiveness to resuscitation, a sudden deterioration for unknown reason, or acute unexplained anemia/blood loss [6]. This protocol provides neonatal practitioners an opportunity to understand the ongoing multiorgan pathophysiology in real time as compared to the conventional blind “guessing” approach that often occurs in the absence of direct physiologic information. The CNP is focused on the evaluation of four vital organs most often compromised in the unstable neonate, as well as an assessment of central line-related complications. The protocol includes (a) Lung-POCUS assessment of pulmonary emergencies (pneumothorax, pleural effusion, or lung atelectasis); (b) Cardiac-POCUS assessment of shock and hemodynamic instability; (c) Cranial-POCUS assessment for acute brain hemorrhage; (d) Abdominal-POCUS assessment of peritoneal or subcapsular bleeding, gut injury, or bowel ischemia; and (e) assessment of central line-related complications by Central-Line POCUS [3, 7].

## Methods

### Steps of developing the consensus statement

**Fig. 1:**
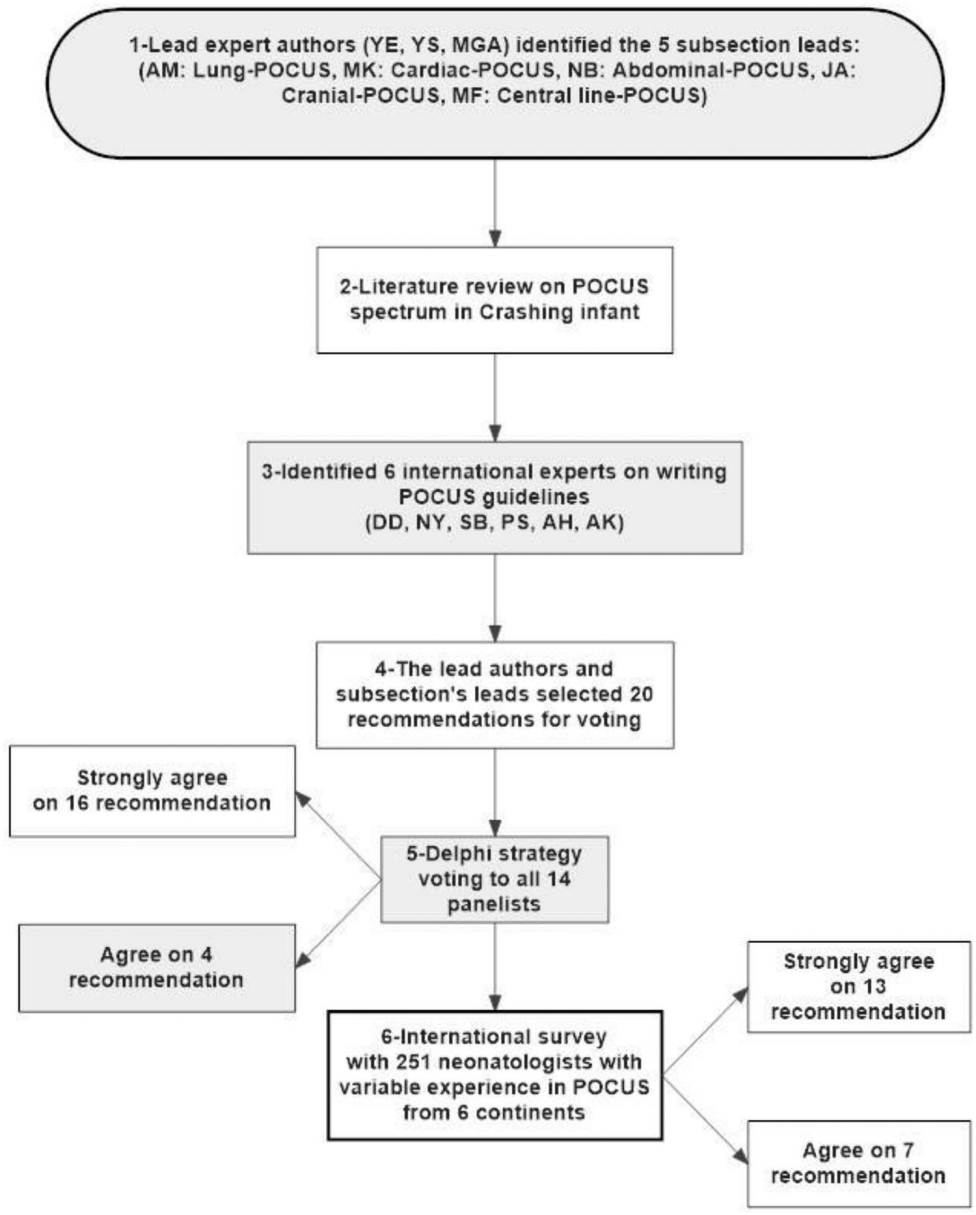
The flow diagram summarizing the six steps applied to reach the consensus agreement

We applied the following six steps in developing the consensus statement, as summarized in Fig. 1:

Step 1: Three lead expert authors in POCUS (YE, YS, and MGA) identified 5 subsection expert leaders (AM: Lung-POCUS, MK: Cardiac-POCUS, NB: Abdominal-POCUS, JA: Cranial-POCUS, MF: Central line-POCUS).
Step 2: The lead authors performed a literature review in the six main domains (general indications of CNP, Lung-POCUS, Cardiac-POCUS, Abdominal-POCUS, Cranial-POCUS, and central line-related complications). The level of evidence was assessed according to the published guidelines (GRADE) [8, 9].
Step 3: The lead authors together with the subsection leaders identified another 6 expert neonatologists who have significantly contributed with institutional guidelines and publications in the field of POCUS and/or developed POCUS training courses in the last 10 years. The selected experts were from Europe, USA, Canada, Asia, and Australia (DD, NY, SB, PS, AH, AK) and together formed a group of 14 panelists.
Step 4: The lead authors and subsection’ leaders selected 20 recommendations for voting. The first 6 recommendations are general indications of the CNP protocol, 4 Lung-POCUS recommendations, 5 Cardiac-POCUS recommendations, 2 Abdominal-POCUS recommendations, one recommendation for each of Cranial and central line-related complications, and one related to the whole algorithm itself.
Step 5: *Step 5*: We applied a modified anonymous electronic Delphi strategy for the online voting process [10]. The Delphi method of voting was among the 14 panelists, each recommendation was graded to 5 grades of agreement: strongly agree, agree, neutral, disagree, and strongly disagree as described in RAND/ULCA published methodology of consensus agreement [11–13].
Step 6: The final step was an open anonymous survey to an international group of 251 neonatologists who are members of the “point of care ultrasound in neonatology association” (pocusneo.org) with a variable degree of experience in utilization of POCUS. The statement recommendations have been prepared according to the international Appraisal of Guidelines, Research and Evaluation (AGREE). Each recommendation is intended to be applied only in the neonatal population with unexplained deterioration as detailed in the protocol [14].

## Steps of developing the CNP

**Fig. 2:**
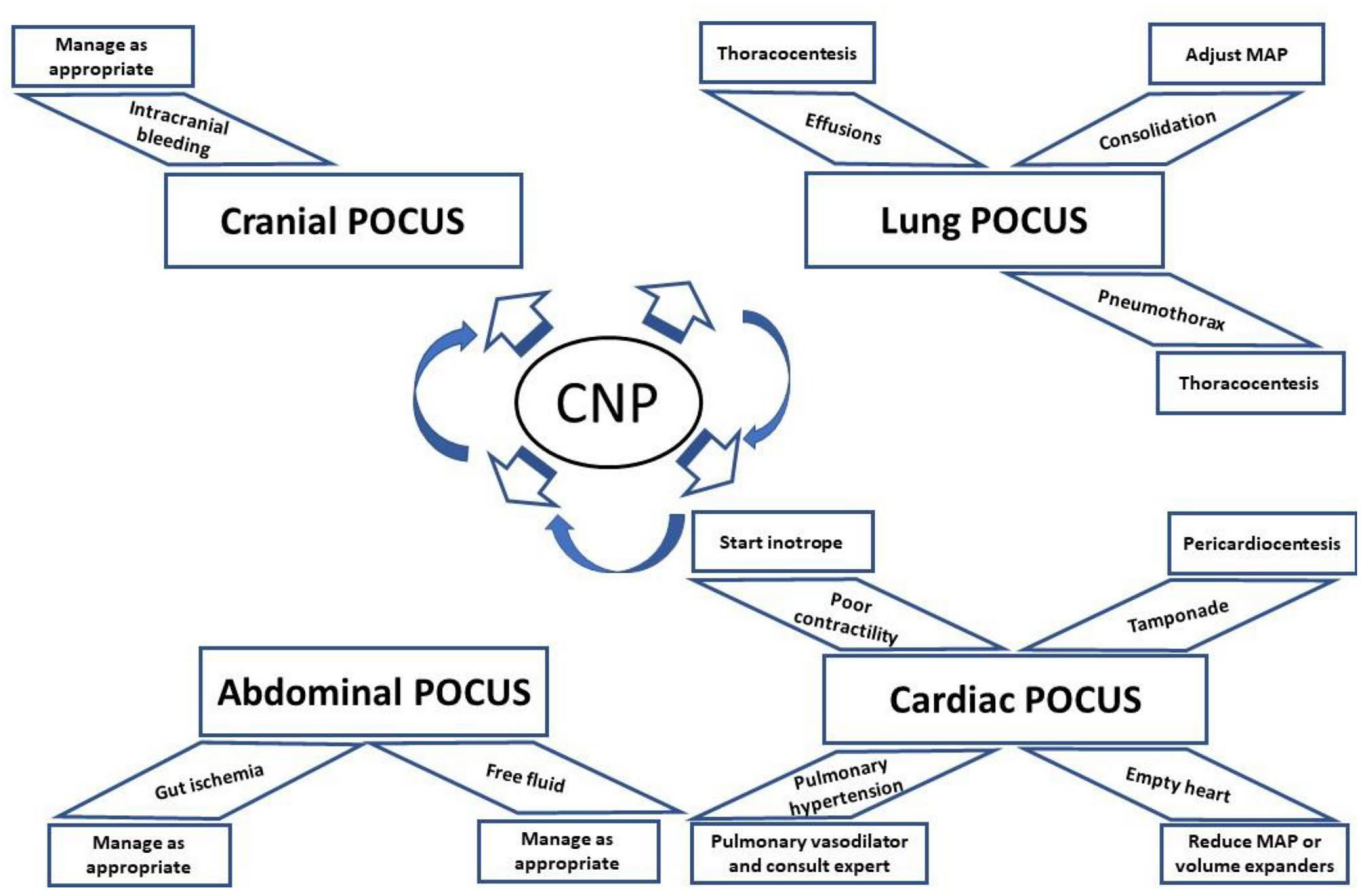
Algorithm for multiorgan systematic assessment by ultrasound for any neonate not responding to the standard steps of resuscitation after birth or any time during NICU admission. The sequence and start point may be different according to the clinical presentation of the crashed or the crashing neonate

The three lead experts met with the subsections leaders and developed the CNP considering the priority steps for assessment of neonate, parallel to neonatal resuscitation program (NRP) [15]. CNP starts with identifying the indication of the protocol as stated in the consensus agreement. The first priority of the NRP is assessment of adequate ventilation and the underlying lung pathology by Lung POCUS and then assessment of circulation by Cardiac POCUS [16, 17–21]. The next step is assessment of cranial hemorrhage by Cranial POCUS [22] followed by abdominal hemorrhage and gut injury on Abdominal POCUS [1, 23]. Then assessment of central line-related complication by Central line POCUS is recommended [24]. The algorithm of the CNP was refined many times before approving the final algorithm described in Fig. 2 by consensus agreement among all authors.

## Results and discussion

A total of 20 recommendations on the use of POCUS in the crashing neonate were assessed. There was strong agreement among all the panelists on 16 recommendations and agreement on 4 recommendations, as detailed in Table 1. We included results of both the Delphi method of voting among the 14 expert panelists and the survey of POCUSNEO members (all members are practicing neonatologists and utilize POCUS in their clinical practice with variable expertise).

**Table 1:** Summary of recommendations for the Crashing Neonate Protocol with levels of agreements and quality of published evidence

POCUS section	Recommendation	Level of agreement		Quality of evidence (GRADE)
		Experts in POCUS *N* = 14	Neonatologists *N* = 251	
General-1	POCUS can provide helpful information when a neonate is not responding to the initial steps of resuscitation	Agree	Strongly agree	C
General-2	POCUS is helpful in evaluating neonates with unexplained circulatory shock	Strongly agree	Strongly agree	C
General-3	POCUS is helpful in evaluating neonates with unexplained lactic acidosis	Strongly agree	Strongly agree	C
General-4	POCUS evaluation is helpful in evaluation of worsening acute hypoxemia unresponsive to routine support	Strongly agree	Strongly agree	C
General-5	POCUS is helpful in assessing neonates with compromised peripheral perfusion, with decreased perfusion index or prolonged capillary refill time	Strongly agree	Strongly agree	C
General-6	POCUS is helpful in localizing a source of hemorrhage when there is an unexplained drop in hematocrit or hemoglobin	Strongly agree	Strongly agree	C
Lung-1	Lung POCUS is helpful to diagnose pneumothorax accurately in the crashing neonate	Strongly agree	Agree	B
Lung-2	Lung POCUS is helpful in diagnosis of pleural effusion in the crashing neonate	Strongly agree	Strongly agree	B
Lung-3	Lung POCUS is helpful in semi quantifying pleural effusion in the crashing neonate	Strongly agree	Strongly agree	D
Lung-4	Lung POCUS is helpful in diagnosis of lung consolidation in the crashing neonate	Strongly agree	Agree	B
Cardiac-1	Cardiac POCUS is helpful in diagnosis of pericardial effusion and pericardial tamponade in the crashing neonate	Strongly agree	Strongly agree	B
Cardiac-2	Cardiac POCUS is helpful in semi-quantification of pericardial effusion in the crashing neonate	Agree	Agree	C
Cardiac-3	Cardiac POCUS is helpful for rapid recognition of poor contractility in the crashing neonate	Agree	Agree	D
Cardiac-4	Cardiac POCUS is helpful in recognition of underfilling of the heart in the crashing neonate	Strongly agree	Strongly agree	D
Cardiac-5	Cardiac POCUS is helpful in recognition of pulmonary hypertension in the crashing neonate	Strongly agree	Agree	B
Cranial	Cranial POCUS is helpful in the assessment of intracranial hemorrhage in neonates with rapidly progressing anemia	Strongly agree	Strongly agree	A
Abdomen-1	Abdominal POCUS is helpful in the diagnosis of ascites or abdominal bleeding in the crashing neonate	Strongly agree	Agree	C
Abdomen-2	Abdominal POCUS is helpful in the diagnosis of gut injury in the crashing neonate	Agree	Agree	B
Line -POCUS	POCUS is helpful in identifying complications related to central lines in the crashing neonate	Strongly agree	Agree	C
Algorithm	Multiorgan assessment (Lung POCUS, Cardiac POCUS, Abdominal POCUS, Cranial POCUS, and Central Line POCUS) as one integrated algorithm is helpful in assessment of the crashing neonate with unknown etiology	Strongly agree	Strongly agree	C

## Recommendations for the general indications of the CNP in neonatal practice

1. *POCUS can provide helpful information when a neonate is not responding to the initial steps of resuscitation—agreement by the panelists and strong agreement by the neonatologists (quality of evidence C)*: When a newborn is not responding to the initial steps of resuscitation (heart rate < 100, low arterial oxygen saturation (SpO2) < 85% after effective positive pressure ventilation adequate oxygen therapy) with no identifiable cause clinically, the underlying cause is secondary to the cardiorespiratory systems in most of the cases as detailed in the next 2 Sects. [10–12].
2. *POCUS is helpful in evaluating infants with unexplained circulatory shock—strong agreement by the panelists and strong agreement by the neonatologists (quality of evidence C)*: In the presence of circulatory shock, defined as blood pressure less than the lower limit for corrected gestational age, POCUS is helpful in understanding the cause and mechanism of shock [26–28].
3. *POCUS is helpful in evaluating infants with unexplained lactic acidosis—strong agreement by the panelists and strong agreement by the neonatologists (quality of evidence C)*: Lactic acidosis is a marker of decreased oxygen delivery which could be due to hemodynamic instability, acute anemia, or severe hypoxia. Identifying the underlying cause can be a challenge without detailed multiorgan assessment using POCUS [25, 28, 29].
4. *POCUS evaluation is helpful in evaluation of worsening acute hypoxemia unresponsive to routine respiratory support—strong agreement by the panelists and strong agreement by the neonatologists (quality of evidence C):* Worsening hypoxemia unresponsive to routine respiratory support and positive pressure ventilation could be secondary to multiple pathophysiologic mechanisms related to parenchymal lung disease and/or cardiac conditions, which may be difficult to diagnose without a detailed assessment using POCUS as explained in lung-POCUS subsection [4, 6, 7].
5. *POCUS is helpful in assessing infants with compromised peripheral perfusion, with decreased perfusion index or prolonged capillary refill time—strong agreement by the panelists and strong agreement by the neonatologists (quality of evidence C)*: Compromised peripheral perfusion can be an early sign of worsening hemodynamic instability or shock, and routine clinical examination has limitations in its ability to diagnose the underlying mechanism [28].
6. *POCUS is helpful in localizing a source of hemorrhage when there is an unexplained drop in hematocrit or hemoglobin—strong agreement by the panelist and strong agreement by the neonatologists (quality of evidence C)*: Perinatal hemorrhage could be secondary to birth trauma, intraventricular hemorrhage in preterm infants, or spontaneous hemorrhage secondary to coagulopathy. Localizing the hemorrhage by ultrasonography is critical for planning medical intervention and is a time-sensitive indication [8–10].

## Recommendations for applying Lung POCUS in the crashing infant

The use of lung ultrasound (LUS) in neonatal and pediatric intensive care has seen rapid growth over the past few years, both for clinical and research purposes [32, 33]. The recently published international guidelines on the use of POCUS provided evidence-based recommendations for diagnosis and monitoring of various lung conditions in critically ill children and neonates [1]. Lung POCUS is an ideal tool for use in emergency situations since it is quick, portable, repeatable, accurate, non-invasive, and radiation free and thereby offers a number of advantages for the clinician when compared with the chest X-ray (CXR) [34].

1. *Lung POCUS is helpful to diagnose pneumothorax accurately in the crashing neonate—strong agreement by the panelists and agreement by the neonatologists (quality of evidence B)*: Evidence from the adult, pediatric, and neonatal literature has shown that LUS has higher diagnostic accuracy (91% sensitivity and 98% specificity) when compared to CXR in detecting pneumothorax, and time to make the diagnosis is shorter [35–37]. Visualization of the following combined LUS patterns can accurately diagnose pneumothorax: (1) absence of “lung sliding sign” of the pleural line, (2) complete absence of B lines, i.e., only A-lines, (3) presence of a “lung point,” and (4) presence of a stratosphere sign on M-mode imaging (Table 2B) [38]. Of note, evidence of pneumothorax on LUS should always be interpreted in the clinical context. Assessment of the lung point, which is the point of separation of the pleural leaflets and is seen at the point where normal sliding pleura meets the non-sliding segment, is specific of pneumothorax and can help in predicting pneumothorax size [36].
2. *Lung POCUS is helpful in diagnosis of pleural effusion in the crashing neonate—strong agreement by the panelists and strong agreement by the neonatologists (quality of evidence B)*: In a newborn with congenital effusion(s), such as those with congenital hydrothorax or chylothorax, may comprise ventilation, and a timely drainage of the effusion is often essential to allow expansion of the lung. Pleural effusion is also an uncommon, but serious complication of central lines, which are one of the mainstays of neonatal critical care in delivering infusions [39].
  Lung POCUS provides a quick and reliable information regarding presence of pleural fluid and has a high diagnostic accuracy, close to that of a CT scan and superior to CXR, with a 93% sensitivity and 96% specificity [40, 41]. The presence of effusion on lung POCUS is seen as a hypoechoic area between the pleural leaflets at the dependent costophrenic angle.
3. *Lung POCUS is helpful in semi-quantifying pleural effusion in the crashing neonate—strong agreement by the panelists and strong agreement by the neonatologists (quality of evidence D)*: Lung POCUS is an excellent tool to rule out or to detect large pleural effusion that may lead to acute decompensation or compromise resuscitation efforts [32, 42, 43]. Although it is easier to diagnose pleural effusion by ultrasound as compared to CXR, the quality of evidence is low on quantifying the volume of pleural effusion in neonates. Hence, for the clinical decision-making, it is better semi-quantified using categories of minimal, small, moderate, or large volume. It is estimated by measuring the distance at the site of maximum collection [40].
4. *Lung POCUS is helpful in diagnosis of lung consolidation in the crashing neonate—strong agreement by the panelists and agreement by the neonatologists (quality of evidence B)*: Lung consolidation is characterized by the presence of a non-aerated area or lung parenchyma filled with fluid [44, 45]. The most common causes are atelectasis, inflammatory processes, severe pulmonary edema, or acute pulmonary hemorrhage [46]. The sonographic appearance of a consolidated lung usually looks like an area with an abnormal pleural line and bronchograms (figure in Table 2A) [30, 47]. Meta-analysis of LUS studies on diagnosis of lung consolidation has shown a high sensitivity and specificity of 96% and 93%, respectively. LUS is superior to both CXR and laboratory tests, even when combined together [48]. In a crashing infant, lung POCUS may help in rapidly diagnosing consolidation and/or atelectasis, which looks like a solid organ-like resembling liver [49, 50].

**Table 2:**
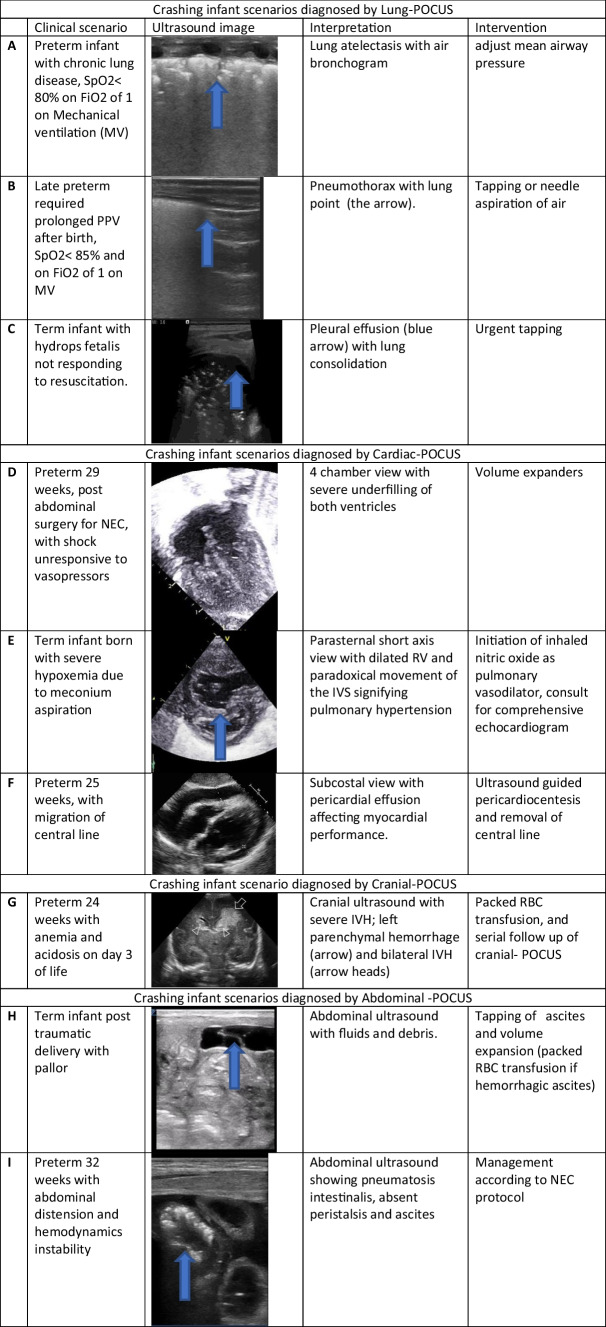
Case scenarios of crashing neonates with the ultrasound images and their interpretation and subsequent interventions

## Recommendations for applying Cardiac POCUS in the crashing neonate

Cardiac POCUS was first described in the 1980s as a readily available, rapid, limited bedside examination performed by emergency physicians to enhance diagnostic capabilities and direct theraphy [51]. In the event of acute decompensation, the goal of cardiac POCUS is to assess cardiac filling and function, pericardial effusion, and ventricular symmetry [52–54]. *Cardiac POCUS is not intended to be used as a screening tool for detection of congenital heart diseases (CHD)* [52]. However, when clinical urgency precludes a comprehensive echocardiographic assessment in a critically ill infant, utilization of standardized protocols by clinicians trained in cardiac POCUS may aid in recognition of abnormalities and help them in consulting a cardiac specialist earlier [1, 7, 55, 56]. CHD, such as outflow tract obstruction, may manifest as poor cardiac function, and recognition of an abnormal cardiac ultrasound may direct management and expedite a comprehensive cardiac evaluation [47].

1. *Cardiac POCUS is helpful in diagnosis of pericardial effusion and cardiac tamponade in the crashing neonate—strong agreement by the panelists and strong agreement by the neonatologists (quality of evidence B)*: One of the most immediate applications of cardiac POCUS in a crashing neonate is to rule out cardiac tamponade or a large pericardial effusion leading to cardiovascular instability [56, 58, 59]. Pericardial effusion can reliably be seen on cardiac POCUS by using multiple echocardiographic views. When cardiac tamponade is detected, POCUS can be used to guide pericardiocentesis, and ultrasound-guided pericardiocentesis is associated with a lower complication rate compared to the traditional landmark technique (figure in Table 2F) [59–61].
2. *Cardiac POCUS is helpful in semi-quantification of pericardial effusion in the crashing neonate*—*agreement by the panelists and agreement by the neonatologists (quality of evidence C)*: The decision to treat pericardial effusion should be made based upon the clinical significance, and ultrasound findings should be interpreted in the clinical context. Nagdev et al. assessed the importance of evaluating the movement of the right ventricle free wall and IVC size during respiratory cycle, and they reported a collapse of right ventricle free wall and absence of inspiratory collapse of IVC as reliable markers to diagnose significant pericardial effusion prior to development of shock [58].
3. *Cardiac POCUS can be used for rapid recognition of poor contractility in the crashing neonate—strong agreement by the panelists and strong agreement by the neonatologists (quality of evidence C)*: International expert consensus statements highlighted the use of cardiac POCUS in pediatrics for assessment of cardiac function and filling [1]. The ability to rapidly identify and address cardiogenic shock can help in earlier initiation of appropriate treatment [62–65]. Qualitative assessment, rather than quantitative measurement of cardiac function from multiple views, including parasternal long and short axes and apical 4-chamber and subcostal view, is one of the primary goals of cardiac POCUS [52]. The use of cardiac POCUS in the hands of non-cardiologist clinicians trained in cardiac ultrasound enables them to differentiate normal and impaired contractility, with good interobserver correlation with cardiologists [66].
4. *Cardiac POCUS is helpful in recognition of underfilling of the heart in the crashing neonate—strong agreement by the panelists and strong agreement by the neonatologists (quality of evidence C)*: The use of clinical findings to guide fluid resuscitation in the neonate is challenging. The traditional markers such as capillary refill, central venous pressure, and tachycardia do not provide a definitive picture of fluid status [67]. The use of cardiac POCUS to evaluate volume status in a critically ill pediatric patient is recommended; however, in the mechanically ventilated neonates, recognition of preload can be challenging, and the findings should be interpreted in the clinical context. Assessment of the difference between end systolic and end diastolic volumes in the apical 4-chamber and parasternal long and short axis views by eyeballing for the filling volumes on 2D and M-Mode is useful for determining the need of fluid resuscitation (Table 2D) [1, 68].
5. *Cardiac POCUS is helpful in recognition of pulmonary hypertension in the crashing neonate—strong agreement by the panelists and agreement by the neonatologists (quality of evidence B)*: In a crashing infant, cardiac POCUS can be used to suspect or rule out moderate to severe pulmonary hypertension [69, 70]. While a detailed assessment of pulmonary hypertension or right ventricular function is out of the scope of cardiac POCUS, it can be utilized for the recognition and a focused evaluation of pulmonary hypertension and assessment of right ventricular function utilizing visual inspection and semi-quantitative assessment [54, 71].

In a crashing infant, pulmonary hypertension can be suspected when there is right ventricular hypertrophy and/or dilation in the presence of clinical suspicion such as persistent hypoxia and significant pre- and post-ductal saturation difference. In the presence of tricuspid regurgitation, pulmonary artery systolic pressure can be reliably estimated using cardiac POCUS [72, 73]. In the absence of tricuspid regurgitation, POCUS can be used for semi-quantitative assessment of pulmonary hypertension by evaluating the interventricular septal position and movement at the end of systole and by assessing the flow direction and velocities across a patent ductus arteriosus and/or foramen ovale (figure in Table 2E) [69]. As stated previously, cardiac POCUS is not a screening tool for CHD, and recognition of abnormality warrants comprehensive cardiac evaluation to ensure a normal structured heart. Infants with suspected or established pulmonary hypertension should have a comprehensive echocardiographic evaluation by the specialist pediatric cardiologist or a neonatologist trained in performing targeted neonatal echocardiography.

## Recommendations for applying Cranial POCUS in the crashing neonate

*Cranial POCUS is helpful in assessment of intracranial hemorrhage in neonates with rapidly progressing anemia—strong agreement by the panelists, and strong agreement by the neonatologists (quality of evidence B)*: Cranial POCUS is the most common neuroimaging modality used in the NICU [62, 63]. In a crashing neonate, cranial POCUS enables the diagnosis of intraventricular, parenchymal, or large cerebellar hemorrhages. Cranial POCUS is usually performed through anterior fontanelle window; however, adding the mastoid fontanelle increases its reliability for diagnosing posterior fossa hemorrhages [74, 75]. Intraventricular hemorrhage (IVH) affects 20–40% of very preterm infants (born before 33 weeks’ gestation) [65, 76]. Extensive IVH or parenchymal hemorrhage may present as a catastrophic event with apnea/bradycardia, hypotension, metabolic acidosis, a rapidly falling hematocrit, or seizures [66, 78]. Cranial POCUS can help in rapidly detecting IVH as per the Volpe or modified Papile classification (figure in Table 2I) [67, 77].

## Recommendations for applying Abdominal POCUS in the crashing neonate

1. *Abdominal POCUS is helpful in the diagnosis of ascites or abdominal bleeding in the crashing neonate—agreement by the panelists, and agreement by the neonatologists (quality of evidence C)*: Ultrasound has a high sensitivity in assessing and localizing abdominal bleeding. While ultrasound cannot differentiate the nature of the fluid, free peritoneal or subcapsular fluid can represent blood in a patient with unexplained anemia or abdominal trauma [31]. Ultrasound can provide information regarding presence of blood in real time and can aid in therapeutic intervention such as ultrasound-guided abdominal paracentesis, making it an excellent tool for use in neonatal emergencies [79, 80].
2. *Abdominal POCUS is helpful in the diagnosis of gut injury in the crashing neonate—agreement by the panelists, agreement by the radiologists, and agreement by the neonatologists (quality of evidence C)*: Ultrasound has been proven as an excellent imaging modality with a high sensitivity in assessing intestinal emergencies including gut ischemia secondary to shock [81, 82]. We recommend assessment of intestinal ischemic injury in any case of shock with unknown cause (figure in Table 2G) [1, 57]. In addition to gut ischemia, abdominal ultrasound can be performed in any infant suspected of having anemic shock due to traumatic abdominal bleeding (figure in Table 2H) [81].

## Recommendations for applying Central Line POCUS in the crashing neonates

*POCUS is helpful in identifying complications related to central lines in the crashing neonate—strong agreement by the panelists and agreement by the neonatologists (quality of evidence C)*: Several studies have questioned the accuracy of X-rays in assessing the line position accurately, reporting a discordance of 20–40% when compared to ultrasound assessment [83]. The anatomical position of the line can be viewed by X-ray, but identifying the intravascular position versus extravascular migration of the line needs either contrast study or ultrasound evaluation. With the available evidence, POCUS may be considered a standard of care for revealing central line tip position and catheter migration [84]. Furthermore, in neonates with acute clinical decompensation with impending cardiac arrest, where pericardial or pleural effusion are suspected due to central line malposition, radiological assessment is insufficient, and POCUS can reliably provide additional information in real time [60, 85, 86].

## Integrated POCUS protocol for the crashing neonate

*Multiorgan assessment (Lung POCUS, Cardiac POCUS, Abdominal POCUS, Cranial POCUS, and Central Line POCUS) as one integrated algorithm is helpful in assessment of the crashing neonate with unknown etiology—strong agreement by the panelists, and strong agreement by the neonatologists (B)*: The proposed protocol introduces a comprehensive approach using POCUS with pre-defined steps focused on the assessment of mechanisms of unresponsiveness to resuscitation, unexplained acute decompensation, or acute unexplained anemia/blood loss. CNP recommends using only focused ultrasound views, which are relatively easy to practice and are reproducible, to detect specific pathologies [5]. Use of a POCUS-guided protocol in evaluation of a sick neonate provides an important opportunity for neonatal practitioners to identify the underlying pathophysiology in a crashing neonate in real time, replacing the empiric approach based upon clinical assessment [84]. This stepwise systematic assessment of the crashing neonate can be applied in any setting such as in the resuscitation room and NICU [5]. However, the sequence and the priority of the organ to be assessed might be different according to the clinical presentation [28]. Although this is the first consensus agreement statement on using POCUS to guide neonatal resuscitation, the practice in using POCUS during adult resuscitation is well established [5, 16].

If there is no response to resuscitation as per the neonatal resuscitation guidelines, we recommend using POCUS to identify the underlying pathology which may explain the reason for unresponsiveness to conventional measures, as demonstrated in the flow diagram (Fig. 2). This stepwise approach encompasses moving from organ to organ, considering the organ priorities as per the Neonatal Resuscitation Program (NRP) [5]. *First*, lung assessment includes assessing lung inflation for optimum ventilation and evaluation for the underlying pathologies such as lung collapse, pneumothorax, effusions [19, 45, 87, 88]. *Second*, cardiac assessment includes underfilling, poor contractility, pulmonary hypertension, and pericardial effusion [58, 89, 90]. If there is associated anemia or severe pallor, or suspicion of line migration, then cranial and abdominal POCUS should also be performed.

We acknowledge the limitations of POCUS-guided proposed protocol including the following: (1) Ultrasound is operator dependent, and the reliability of the images depends on the training and competency of the operator, quality of images, and the machine; (2) the operator needs to be trained in multiorgan assessment with completion of acceptable training in each module, so a clear institutional protocol for training and guidelines for practice should be considered; (3) CNP is time-sensitive and ideally needs a trained person being available in the NICU at any time; and (4) the level of evidence supporting the crashing neonate protocol is low to intermediate level (mostly B to C grade), and most studies supporting POCUS use are either observational or retrospective analysis type. An important next step would be to formally study the learning curve needed to apply the CNP protocol as a whole and/or each organ section.

## Conclusion

The newly proposed protocol for the crashing neonate can be used as an adjunct to the current recommendations for neonatal resuscitation. The CNP protocol is proposed based upon preidentified steps focused on gaining information regarding pathophysiology in infants with unexplained clinical deterioration or those not responding to the standard resuscitation. In comparison to the currently used diagnostic aids, POCUS can provide valuable information in real time to answer a specific question relating to diagnosis or ruling out potential causes of deterioration. It can help in early diagnosis and facilitates targeting of specific intervention based upon the underlying pathophysiology in neonates, similar to the well-established application in pediatric and adult clinical practice.

## Data Availability

All data and original material are available for transparency.

## Abbreviations

AXR: Abdominal X-ray
CHD: Congenital heart disease
CNP: Crashing Neonate Protocol
CXR: Chest X-ray
LUS: Lung ultrasound
NICU: Neonatal intensive care unit
POCUS: Point-of-care ultrasound

## References

[1] Singh Y, Tissot C, Fraga MV, Yousef N, Cortes RG, Lopez J (2020). International evidence-based guidelines on point of care ultrasound (POCUS) for critically ill neonates and children issued by the POCUS Working Group of the European Society of Paediatric and Neonatal Intensive Care (ESPNIC). Crit Care.

[2] Nguyen J, Amirnovin R, Ramanathan R, Noori S (2016) The state of point-of-care ultrasonography use and training in neonatal–perinatal medicine and pediatric critical care medicine fellowship programs. J Perinatol 36(11):972–6. Available from: http://www.nature.com/doifinder/10.1038/jp.2016.126 10.1038/jp.2016.126 27513327

[3] Miller LE, Stoller JZ, Fraga MV (2020). Point-of-care ultrasound in the neonatal ICU. Curr Opin Pediatr.

[4] Raimondi F, Yousef N, Migliaro F, Capasso L, De Luca D (2021) Point-of-care lung ultrasound in neonatology: classification into descriptive and functional applications. Pediatr Res 90(3):524–31. Available from: 10.1038/s41390-018-0114-9 10.1038/s41390-018-0114-9 PMC7094915 30127522

[5] Yousef N, Singh Y, De Luca D (2021) Playing it SAFE in the NICU SAFE-R: a targeted diagnostic ultrasound protocol for the suddenly decompensating infant in the NICU. Eur J Pediatr 0123456789 10.1007/s00431-021-04186-w PMC8256195 34223967

[6] Conlon TW, Nishisaki A, Singh Y, Bhombal S, De Luca D, Kessler DO et al (2019) Moving beyond the stethoscope: diagnostic point-of-care ultrasound in pediatric practice. Pediatrics 144(4) 10.1542/peds.2019-1402 31481415

[7] Singh Y, Tissot C (2018) Echocardiographic evaluation of transitional circulation for the neonatologists. Front Pediatr 6(May):140. Available from: http://journal.frontiersin.org/article/10.3389/fped.2018.00140/full 10.3389/fped.2018.00140 PMC5962801 29868528

[8] Meader N, King K, Llewellyn A, Norman G, Brown J, Rodgers M (2014). A checklist designed to aid consistency and reproducibility of GRADE assessments: development and pilot validation. Syst Rev.

[9] Volpicelli G, Elbarbary M, Blaivas M, Lichtenstein DA, Mathis G, Kirkpatrick AW (2012). International evidence-based recommendations for point-of-care lung ultrasound. Intensive Care Med.

[10] Gnatzy T, Warth J, von der Gracht H, Darkow IL (2011) Validating an innovative real-time Delphi approach - a methodological comparison between real-time and conventional Delphi studies. Technol Forecast Soc Change 78(9):1681–94. Available from: 10.1016/j.techfore.2011.04.006

[11] De Luca D, van Kaam AH, Tingay DG, Courtney SE, Danhaive O, Carnielli VP et al (2017) The Montreux definition of neonatal ARDS: biological and clinical background behind the description of a new entity. Lancet Respir Med 5(8):657–66. Available from: 10.1016/S2213-2600(17)30214-X 10.1016/S2213-2600(17)30214-X 28687343

[12] De Luca D, Cogo P, Kneyber MC, Biban P, Semple MG, Perez-Gil J et al (2021) Surfactant therapies for pediatric and neonatal ARDS: ESPNIC expert consensus opinion for future research steps. Crit Care 25(1):1–12. Available from: 10.1186/s13054-021-03489-6 10.1186/s13054-021-03489-6 PMC7898495 33618742

[13] Teoh AYB, Dhir V, Kida M, Yasuda I, Jin ZD, Seo DW (2018). Consensus guidelines on the optimal management in interventional EUS procedures: results from the Asian EUS group RAND/UCLA expert panel. Gut.

[14] Brouwers MC, Kerkvliet K, Spithof K (2016) The AGREE reporting checklist: a tool to improve reporting of clinical practice guidelines. BMJ 352 10.1136/bmj.i1152 PMC5118873 26957104

[15] Zaichkin J, Kamath-Rayne BD, Weiner G (2021). The NRP 8th edition: innovation in education. Neonatal Netw.

[16] Blank DA, Rogerson SR, Kamlin COF, Fox LM, Lorenz L, Kane SC et al (2017) Lung ultrasound during the initiation of breathing in healthy term and late preterm infants immediately after birth, a prospective, observational study. Resuscitation 114:59–65. Available from: 10.1016/j.resuscitation.2017.02.017 10.1016/j.resuscitation.2017.02.017 28249708

[17] Reynolds R, Pilcher J, Ring a, Johnson R, McKinley P (2009) The golden hour: care of the LBW infant during the first hour of life one unit’s experience. Neonatal Netw 28(4):211–9. Available from: http://search.ebscohost.com/login.aspx?direct=true&db=c8h&AN=2010342856&lang=es&site=ehost-live 10.1891/0730-0832.28.4.211 19592362

[18] Brat R, Yousef N, Klifa R, Reynaud S, Shankar Aguilera S, De Luca D (2015) Lung ultrasonography score to evaluate oxygenation and surfactant need in neonates treated with continuous positive airway pressure. JAMA Pediatr 169(8):e151797. Available from: http://archpedi.jamanetwork.com/article.aspx?doi=10.1001/jamapediatrics.2015.1797 10.1001/jamapediatrics.2015.1797 26237465

[19] Raimondi F, Rodriguez Fanjul J, Aversa S, Chirico G, Yousef N, De Luca D et al (2015) Lung ultrasound for diagnosing pneumothorax in the critically ill neonate. J Pediatr 10.1016/j.jpeds.2016.04.018 27189678

[20] De Martino L, Yousef N, Ben-Ammar R, Raimondi F, Shankar-Aguilera S, De Luca D (2018). Lung ultrasound score predicts surfactant need in extremely preterm neonates. Pediatrics.

[21] Seri I (2001). Circulatory support of the sick preterm infant. Semin Neonatol.

[22] van Wezel-Meijler G, Steggerda SJ, Leijser LM (2010) Cranial ultrasonography in neonates: role and limitations. Semin Perinatol 34(1):28–38. Available from: 10.1053/j.semperi.2009.10.002 10.1053/j.semperi.2009.10.002 20109970

[23] Bohnhorst B (2013) Usefulness of abdominal ultrasound in diagnosing necrotising enterocolitis. Arch Dis Child Fetal Neonatal Ed 98:F445–50. Available from: http://www.ncbi.nlm.nih.gov/pubmed/23572342 10.1136/archdischild-2012-302848 23572342

[24] Ramasethu J (2008). Complications of vascular catheters in the neonatal intensive care unit. Clin Perinatol.

[25] Atkinson P, Bowra J, Milne J, Lewis D, Lambert M, Jarman B (2017). International Federation for emergency medicine consensus statement: sonography in hypotension and cardiac arrest (SHoC): an international consensus on the use of point of care ultrasound for undifferentiated hypotension and during cardiac arrest. Can J Emerg Med.

[26] Marin JR, Abo AM, Arroyo AC, Doniger SJ, Fischer JW, Rempell R et al (2016) Pediatric emergency medicine point-of-care ultrasound: summary of the evidence. Crit Ultrasound J 8(1) 10.1186/s13089-016-0049-5 PMC5095098 27812885

[27] Elsayed YN, Amer R, Seshia MM (2017) The impact of integrated evaluation of hemodynamics using targeted neonatal echocardiography with indices of tissue oxygenation: a new approach. J Perinatol 10.1038/jp.2016.257 28102856

[28] Elsayed Y, Abdul Wahab MG (2021) A new physiologic-based integrated algorithm in the management of neonatal hemodynamic instability. Eur J Pediatr. Available from: 10.1007/s00431-021-04307-5 10.1007/s00431-021-04307-5 34748080

[29] Elsayed Y, Abdelmawla M, Narvey M, Wrogemann J (2017) A model of integrated lung and focused heart ultrasound as a new screening examination in infants at risk of respiratory or hemodynamic compromise. J Pediatr Neonatal Individ Med 6(1)

[30] Lichtenstein DA (2015) BLUE-Protocol and FALLS-Protocol. Chest 147(6):1659–70. Available from: http://linkinghub.elsevier.com/retrieve/pii/S0012369215372238 10.1378/chest.14-1313 26033127

[31] Descamps CS, Cneude F, Hays S, Rayet I, Piolat C, Epiard C (2017). Early hypovolemic shock and abdominal distention due to neonatal splenic rupture: urgency of diagnosis and management. Eur J Pediatr.

[32] Staub LJ, Biscaro RRM, Kaszubowski E, Maurici R (2018). Chest ultrasonography for the emergency diagnosis of traumatic pneumothorax and haemothorax: a systematic review and meta-analysis. Injury.

[33] Raimondi F, Yousef N, Migliaro F, Capasso L, De Luca D (2021) Point-of-care lung ultrasound in neonatology: classification into descriptive and functional applications . Vol. 90, Pediatric Research. Springer US 524–31. Available from: 10.1038/s41390-018-0114-9 10.1038/s41390-018-0114-9 PMC7094915 30127522

[34] Escourrou G, De Luca D (2016). Lung ultrasound decreased radiation exposure in preterm infants in a neonatal intensive care unit. Acta Paediatr Int J Paediatr.

[35] Cattarossi L, Copetti R, Brusa G, Pintaldi S (2016) Lung ultrasound diagnostic accuracy in neonatal pneumothorax. 2016(1) 10.1155/2016/6515069 PMC4904536 27445558

[36] Volpicelli G, Boero E, Sverzellati N, Cardinale L, Busso M, Boccuzzi F (2014). Semi-quantification of pneumothorax volume by lung ultrasound. Intensive Care Med.

[37] Collins CD, Lopez A, Mathie A, Wood V, Jackson JE, Roddie ME (1995) Quantification of pneumothorax size on chest radiographs using interpleural distances: regression analysis based on volume measurements from helical CT. Am J Roentgenol 165(5):1127–30 10.2214/ajr.165.5.7572489 7572489

[38] Elsayed YN, Hinton M, Graham R, Dakshinamurti S (2020) Lung ultrasound predicts histological lung injury in a neonatal model of acute respiratory distress syndrome. Pediatr Pulmonol 1–11 10.1002/ppul.24993 PMC7436735 32741109

[39] Shih YT, Su PH, Chen JY, Lee IC, Hu JM, Chang HP (2011) Common etiologies of neonatal pleural effusion. Pediatr Neonatol 52(5):251–5. Available from: 10.1016/j.pedneo.2011.06.002 10.1016/j.pedneo.2011.06.002 22036219

[40] Grimberg A, Shigueoka DC, Atallah ÁN, Ajzen S, Iared W (2010). Diagnostic accuracy of sonography for pleural effusion: systematic review. Sao Paulo Med J.

[41] Laursen CB, Clive A, Hallifax R, Pietersen PI, Asciak R, Davidsen JR et al (2021) European Respiratory Society statement on thoracic ultrasound. Eur Respir J 57(3):1–26. Available from: 10.1183/13993003.01519-2020 10.1183/13993003.01519-2020 33033148

[42] Kocijančič I, Vidmar K, Ivanovi-Herceg Z (2003). Chest sonography versus lateral decubitus radiography in the diagnosis of small pleural effusions. J Clin Ultrasound.

[43] Rocco M, Carbone I, Morelli A, Bertoletti L, Rossi S, Vitale M (2008). Diagnostic accuracy of bedside ultrasonography in the ICU: feasibility of detecting pulmonary effusion and lung contusion in patients on respiratory support after severe blunt thoracic trauma. Acta Anaesthesiol Scand.

[44] Liu J, Chen XX, Li XW, Chen SW, Yan W, Fu W (2016) Lung ultrasonography to diagnose transient tachypnea of the newborn. Chest 149(5):1269–75. Available from: 10.1016/j.chest.2015.12.024 10.1016/j.chest.2015.12.024 26836942

[45] Lichtenstein DA, Mauriat P (2012) Lung ultrasound in the critically ill neonate. Curr Pediatr Rev 8(3):217–23 10.2174/157339612802139389 PMC3522086 23255876

[46] Ren XL, Fu W, Liu J, Liu Y, Xia RM (2017). Lung ultrasonography to diagnose pulmonary hemorrhage of the newborn. J Matern Neonatal Med.

[47] Xirouchaki N, Magkanas E, Vaporidi K, Kondili E, Plataki M, Patrianakos A et al (2011) Lung ultrasound in critically ill patients: comparison with bedside chest radiography. Intensive Care Med 10.1007/s00134-011-2317-y 21809107

[48] Pereda M a., Chavez M a., Hooper-Miele CC, Gilman RH, Steinhoff MC, Ellington LE, et al. Lung ultrasound for the diagnosis of pneumonia in children: a meta-analysis. Pediatrics [Internet]. 2015;135(4):714–22. Available from: http://pediatrics.aappublications.org/cgi/doi/10.1542/peds.2014-2833 10.1542/peds.2014-2833 PMC9923609 25780071

[49] Reports from M (2021) Piastra and co-researchers add new data to findings in human development (lung ultrasound findings in meconium aspiration syndrome) 10.1016/S0378-3782(14)50011-4 25220126

[50] Loi B, Vigo G, Baraldi E, Raimondi F, Carnielli VP, Mosca F et al (2020) Lung ultrasound to monitor extremely preterm infants and predict bpd: multicenter longitudinal cohort study. Am J Respir Crit Care Med 1–52 10.1164/rccm.202008-3131OC 33352083

[51] Mayron R, Gaudio FE, Plummer D, Asinger R (2014) Echocardiography performed by emergency physicians: impact on diagnosis and therapy. 1–5. Available from: https://papers2://publication/uuid/631BE5FF-37F6–4A39-BD51-F01D127AFA36 10.1016/s0196-0644(88)80301-9 3337431

[52] Mertens L, Seri I, Marek J, Arlettaz R, Barker P, McNamara P et al (2011) Targeted neonatal echocardiography in the neonatal intensive care unit: practice guidelines and recommendations for training. Eur J Echocardiogr [cited 2012 Jun 25] 12(10):715–36. Available from: http://www.ncbi.nlm.nih.gov/pubmed/21998460 10.1093/ejechocard/jer181 21998460

[53] Evans N, Gournay V, Cabanas F, Kluckow M, Leone T, Groves A et al (2011) Point-of-care ultrasound in the neonatal intensive care unit: international perspectives. Semin Fetal Neonatal Med [Internet]. Feb [cited 2012 Jul 11] 16(1):61–8. Available from: http://www.ncbi.nlm.nih.gov/pubmed/20663724 10.1016/j.siny.2010.06.005 20663724

[54] Jain A, Mohamed A, El-Khuffash A, Connelly KA, Dallaire F, Jankov RP (2014). A comprehensive echocardiographic protocol for assessing neonatal right ventricular dimensions and function in the transitional period: normative data and z scores. J Am Soc Echocardiogr.

[55] Singh Y, Katheria AC, Vora F (2018). Advances in diagnosis and management of hemodynamic instability in neonatal shock. Front Pediatr.

[56] Singh Y (2017). Echocardiographic evaluation of hemodynamics in neonates and children. Front Pediatr.

[57] Słodki M, Respondek-Liberska M, Pruetz JD, Donofrio MT (2016) Fetal cardiology: changing the definition of critical heart disease in the newborn. J Perinatol 1–6. Available from: http://www.nature.com/doifinder/10.1038/jp.2016.20 10.1038/jp.2016.20 26963427

[58] Nagdev A, Stone MB (2011) Point-of-care ultrasound evaluation of pericardial effusions : does this patient have cardiac tamponade ? 82:671–3 10.1016/j.resuscitation.2011.02.004 21397379

[59] Longjohn M, Pershad J (2011). Point-of-care echocardiography by pediatric emergency physicians. Clin Pediatr Emerg Med.

[60] Labovitz AJ, Noble VE, Bierig M, Goldstein SA, Jones R, Kort S et al (2010) Focused cardiac ultrasound in the emergent setting: a consensus statement of the American Society of Echocardiography and American College of Emergency Physicians. J Am Soc Echocardiogr 23(12):1225–30. Available from: 10.1016/j.echo.2010.10.005 10.1016/j.echo.2010.10.005 21111923

[61] Ma IWY, Arishenkoff S, Wiseman J, Desy J, Ailon J, Martin L (2017). Internal medicine point-of-care ultrasound curriculum: consensus recommendations from the Canadian Internal Medicine Ultrasound (CIMUS) Group. J Gen Intern Med.

[62] Giesinger RE, Elsayed YN, Castaldo MP, McNamara PJ (2019) Targeted neonatal echocardiography-guided therapy in vein of Galen aneurysmal malformation: a report of two cases with a review of physiology and approach to management. AJP Rep 9(2) 10.1055/s-0039-1688765 PMC6541491 31149387

[63] Finan E, Sehgal A, Khuffash AE, McNamara PJ (2014) Targeted neonatal echocardiography services: need for standardized training and quality assurance. J Ultrasound Med 33(10):1833–41. Available from: http://www.jultrasoundmed.org//cgi/doi/10.7863/ultra.33.10.1833 10.7863/ultra.33.10.1833 25253831

[64] Harabor A, Soraisham AS (2015) Utility of targeted neonatal echocardiography in the management of neonatal illness. J Ultrasound Med 34(7):1259–63. Available from: http://doi.wiley.com/10.7863/ultra.34.7.1259 10.7863/ultra.34.7.1259 26112629

[65] Noori S, Seri I (2014) Does targeted neonatal echocardiography affect hemodynamics and cerebral oxygenation in extremely preterm infants? J Perinatol 10.1038/jp.2014.127 25033075

[66] Corredera a, Rodríguez MJ, Arévalo P, Llorente B, Moro M, Arruza L (2014) [Functional echocardiography in neonatal intensive care: 1 year experience in a unit in Spain]. An Pediatr (Barc) 81(3):167–73. Available from: http://www.sciencedirect.com/science/article/pii/S1695403313004931 10.1016/j.anpedi.2013.11.026 24387937

[67] Giesinger RE, McNamara PJ (2016) Hemodynamic instability in the critically ill neonate: an approach to cardiovascular support based on disease pathophysiology. Semin Perinatol 40(3):174–88. Available from: 10.1053/j.semperi.2015.12.005 10.1053/j.semperi.2015.12.005 26778235

[68] Yamamoto K, Nishimura RA, Burnett JC, Redfield MM (1997). Assessment of left ventricular end-diastolic pressure by Doppler echocardiography: contribution of duration of pulmonary venous versus mitral flow velocity curves at atrial contraction. J Am Soc Echocardiogr.

[69] Jain A (2013). Mcnamara PJ. Persistent pulmonary hypertension of the newborn : physiology, hemo-dynamic assessment and novel therapies.

[70] Abman SH (2016) New guidelines for managing pulmonary hypertension. Curr Opin Pediatr 28(5):597–606. Available from: http://content.wkhealth.com/linkback/openurl?sid=WKPTLP:landingpage&an=00008480-201610000-00006 10.1097/MOP.0000000000000403 PMC5094361 27583409

[71] El-Khuffash a, Herbozo C, Jain a, Lapointe A, McNamara PJ (2013) Targeted neonatal echocardiography (TnECHO) service in a Canadian neonatal intensive care unit: a 4-year experience. J Perinatol 33(February):1–4. Available from: http://www.ncbi.nlm.nih.gov/pubmed/23619373 10.1038/jp.2013.42 23619373

[72] Amer R, Elsayed YN, Graham MR, Sikarwar AS, Hinton M, Dakshinamurti S (2019) Effect of vasopressin on a porcine model of persistent pulmonary hypertension of the newborn. Pediatr Pulmonol 54(3) 10.1002/ppul.24248 30644649

[73] Giesinger RE, Elsayed YN, Castaldo MP, McNamara PJ (2019). Targeted neonatal echocardiography-guided therapy in vein of Galen aneurysmal malformation: a report of two cases with a review of physiology and approach to management. AJP Rep.

[74] Correa F, Enrı G (2004) Posterior fontanelle sonography : an acoustic window into the neonatal brain. 1274–82 PMC7976539 15313724

[75] Brouwer MJ, Benders MJNL (2012) Pediatric Imaging New reference values for the neonatal cerebral ventricles 1 Purpose : Methods : Results. 262(1) 10.1148/radiol.11110334 22084208

[76] Linder N, Haskin O, Levit O, Klinger G, Prince T, Naor N et al (2003) Risk factors for intraventricular hemorrhage in very low birth weight premature infants: a retrospective case-control study. Pediatrics 111(5 Pt 1):e590–5. Available from: http://www.ncbi.nlm.nih.gov/pubmed/12728115 10.1542/peds.111.5.e590 12728115

[77] Papile LA, Burstein J, Burstein R, Koffler H (1978). Incidence and evolution of subependymal and intraventricular hemorrhage: a study of infants with birth weights less than 1,500 gm. J Pediatr.

[78] Noori S, McCoy M, Anderson MP, Ramji F, Seri I (2014) Changes in cardiac function and cerebral blood flow in relation to peri/intraventricular hemorrhage in extremely preterm infants. J Pediatr 164(2):264–70.e1–3. Available from: http://www.ncbi.nlm.nih.gov/pubmed/24183212 10.1016/j.jpeds.2013.09.045 24183212

[79] Ecury-goossen GM, Camfferman FA, Leijser LM, Govaert P, Brierley J, Colunga JM et al (2020) evidence-based guidelines on point of care ultrasound ( POCUS ) for critically ill neonates and children issued by the POCUS Working Group of the European Society of Paediatric and. 21(4):2006–8 10.1186/s13054-020-2787-9 PMC7041196 32093763

[80] Becker BA, Lahham S, Gonzales MA, Nomura JT, Bui MK, Truong TA (2019). A prospective, multicenter evaluation of point-of-care ultrasound for small-bowel obstruction in the emergency department. Acad Emerg Med.

[81] Elsayed Y, Seshia M (2022) A new intestinal ultrasound integrated approach for the management of neonatal gut injury. Eur J Pediatr 0123456789. Available from: 10.1007/s00431-021-04353-z 10.1007/s00431-021-04353-z 34981184

[82] Gale HI, Gee MS, Westra SJ, Nimkin K (2016) Abdominal ultrasonography of the pediatric gastrointestinal tract. World J Radiol 8(7):656. Available from: http://www.wjgnet.com/1949-8470/full/v8/i7/656.htm 10.4329/wjr.v8.i7.656 PMC4965350 27551336

[83] Nguyen J (2016). Ultrasonography for central catheter placement in the neonatal intensive care unit - a review of utility and practicality. Am J Perinatol.

[84] Fraga MV, Stoller JZ, Glau CL, De Luca D, Rempell RG, Wenger JL et al (2019) Seeing is believing: ultrasound in pediatric procedural performance. Pediatrics 144(5) 10.1542/peds.2019-1401 31615954

[85] Katheria a C, Fleming SE, Kim JH (2013) A randomized controlled trial of ultrasound-guided peripherally inserted central catheters compared with standard radiograph in neonates. J Perinatol 33(10):791–4. Available from: http://www.ncbi.nlm.nih.gov/pubmed/23765173 10.1038/jp.2013.58 23765173

[86] Vieillard-Baron A, Slama M, Cholley B, Janvier G, Vignon P (2008) Echocardiography in the intensive care unit: from evolution to revolution? Intensive Care Med [cited 2012 Mar 18] 34(2):243–9. Available from: http://www.ncbi.nlm.nih.gov/pubmed/17992511 10.1007/s00134-007-0923-5 17992511

[87] Liu J (2014) Lung ultrasonography for the diagnosis of neonatal lung disease. J Matern Fetal Neonatal Med 27(8):856–61. Available from: http://eutils.ncbi.nlm.nih.gov/entrez/eutils/elink.fcgi?dbfrom=pubmed&id=24028601&ret mode=ref&cmd=prlinks%5Cnpapers3://publication/doi/10.3109/14767058.2013.844125 10.3109/14767058.2013.844125 24028601

[88] Bouhemad B, Brisson H, Le-Guen M, Arbelot C, Lu Q, Rouby JJ (2011). Bedside ultrasound assessment of positive end-expiratory pressure-induced lung recruitment. Am J Respir Crit Care Med.

[89] Labovitz AJ, Noble VE, Bierig M, Goldstein SA, Jones R, Kort S et al (2010) Focused cardiac ultrasound in the emergent setting: a consensus statement of the American society of Echocardiography and American College of Emergency Physicians. J Am Soc Echocardiogr 23(12):1225–30. Available from: 10.1016/j.echo.2010.10.005 10.1016/j.echo.2010.10.005 21111923

[90] Via G, Hussain A, Wells M, Reardon R, Elbarbary M, Noble VE (2014). International evidence-based recommendations for focused cardiac ultrasound. J Am Soc Echocardiogr.

